# Zintl Phase versus Covalent Metal: Chemical Bonding in Silicon Dumbbells of Ca_5_Si_3_ and CaSi_3_

**DOI:** 10.1021/acs.inorgchem.4c01464

**Published:** 2024-06-24

**Authors:** Kati Finzel, Ulrich Schwarz

**Affiliations:** Max Planck Institute for Chemical Physics of Solids Noethnitzer Str. 40, 01187 Dresden, Germany

## Abstract

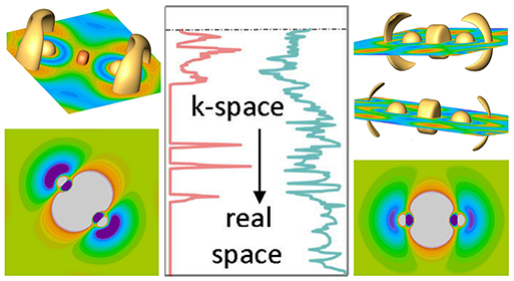

Silicon dumbbells constitute identifiable anionic molecular species in Zintl phases and so-called covalent metals holding units with homopolar bonding inside a metallic framework. Based on electron-precise Ca_5_Si_3_ and metallic CaSi_3_, the chemical bonding in Si_2_ units is investigated by computational quantum chemical methods considering the dual nature of the wave function. This concerted wave-vector and real space study substantiates that the Si_2_ dumbbells in Ca_5_Si_3_ can be referred to as molecular building units Si_2_^6–^ with additional metallic and ionic contributions in the solid. In the covalent metal CaSi_3_, however, the bonding within the dumbbells falls short of fulfilling the octet rule. As a result, antibonding states of the Si_2_ building units are depopulated and attend metallic interactions, simultaneously giving rise to stronger covalent Si–Si bonds.

## Abstract 2

The density of states of electron-precise Ca_5_Si_3_ exhibits sharp maxima of the valence states while CaSi_3_ shows a metal-like dispersion in the valence region. The difference in *k*-space corresponds to dissimilar bonding situations as evidenced by real-space techniques. The results evidence classical closed-shell molecular anions in Ca_5_Si_3_ as compared to reduced population of antibonding states in CaSi_3_ giving rise to stronger Si−Si interactions in the dumbbells.

## Introduction

The probably most prominent achievement of Eduard Zintl’s studies is the classification of alloys and the identification of geometrical and electronic factors governing the structural organization of ordered intermetallic compounds, a cornerstone for chemists’ present day understanding of electron-precise alloy phases.^1^ The stepwise development of the Zintl-concept started out by establishing crystal chemical criteria for distinguishing salt-like and alloy-type atomic arrangements of strongly polar intermetallic phases.^2^ Using the example of binary magnesium compounds,^3^ the classification is based on the presence or absence of homonuclear contacts in the coordination environment of the constituting elements. Based on the findings of detailed structural investigations, Zintl strived to comprehend the ability of p- and d-block elements to form anions in the compounds with the less electronegative members of the s-block, i.e., alkali or alkaline-earth metals.^4^ Careful and, at that time, advanced studies analyzed the effect of the formal charge transfer on atomic radii and chemical bonding.^5,6^ By way of example, the compound LiAl adopts a NaTl atomic arrangement in which the aluminum accepts an electron of lithium. The resulting Al^–^ polyanion holding four valence electrons per atom then forms the very same diamond-type substructure as the isoelectronic element silicon.^6^ Subsequent researchers expanded the original concept significantly into the current model of bonding in electron-precise intermetallic compounds.^7−11^ Expert discussions of the various aspects of the Zintl concept have been given in recent comprehensive reviews.^12−34^

Within the extended class of Zintl phases, silicon is one of the p-block elements which forms an outstandingly large variety of anionic species and structural patterns,^11^ e.g., in the binary system Ca–Si. Isolated Si^4–^ ions occur in Ca_2_Si and Ca_5_Si_3_,^35,36^ the latter additionally comprising silicon dumbbells of one-bonded Si^3–^ anions. Two-bonded Si^2–^ ions forming zigzag chains are present in CaSi,^37,38^ and three-bonded anions Si^–^ establish puckered nets in modifications of CaSi_2_.^39−41^ More complex Zintl anions with silicon in different bonding situations are present in Ca_3_Si_4_ and Ca_14_Si_19_.^42,43^

Assuming full charge transfer from calcium to silicon, the number of bonds in all anionic partial structures is in-line with the 8 – *N* rule. The formal description is supported by a concerted theoretical and experimental study using the example of CaSi.^38^ The seminal investigation confirms covalent bonding in the zigzag chains constituted by silicon anions. The species Si^2–^ is valence isoelectronic to sulfur and forms two homopolar bonds in essential agreement with the Zintl concept. Nevertheless, the authors find evidence for additional nonionic interactions between calcium and silicon that remain unaccounted for within the framework of the Zintl concept.^38^

In the last decades, the already rich diversity of alkaline-earth metal compounds holding silicon polyanionic building units has been significantly extended by explorative application of high-pressure techniques.^44−48^ The phases provide access to atomic arrangements in which the chemical bonding can no longer be described within the framework of the 8 – *N* rule. Such a situation typically gives rise to metallic properties, and in conjunction with homopolar bonding in the polyanions, numerous so-called covalent metals are formed.

An elementary structure motif occurring in Zintl-phases as well as in covalent metals is the Si_2_ dumbbell. For the present study, we selected Ca_5_Si_3_ and CaSi_3_ as prototype examples, which also allow for comparison with the findings of earlier studies on various electron-precise calcium silicides.^38,49−53^ We aim at giving a combined picture of chemical bonding using well-established indicators from solid-state theory together with tools from real space analysis, i.e., methods that extract unitary invariant indicators in three-dimensional real space for bonding analysis from the multidimensional wave function rather than analyzing wave-vector-based quantities or orbitals. Both methods operate in complementary representations, i.e., the DOS depicts the number of electrons in a given energy window while the electron density, and ELI-D alike, yield the electronic charge in a defined volume in position space. The respective findings, however, must converge to a congruent picture as they are based on the same wave function. Therefore, changes in so-called *k*-space will also provide signatures in real space and vice versa. In this study, we aim at emphasizing this dualism aspect for the interpretation of chemical bonding in simple diatomic silicon units.

## Results and Discussion

### Crystal Structures

**Figure 1:**
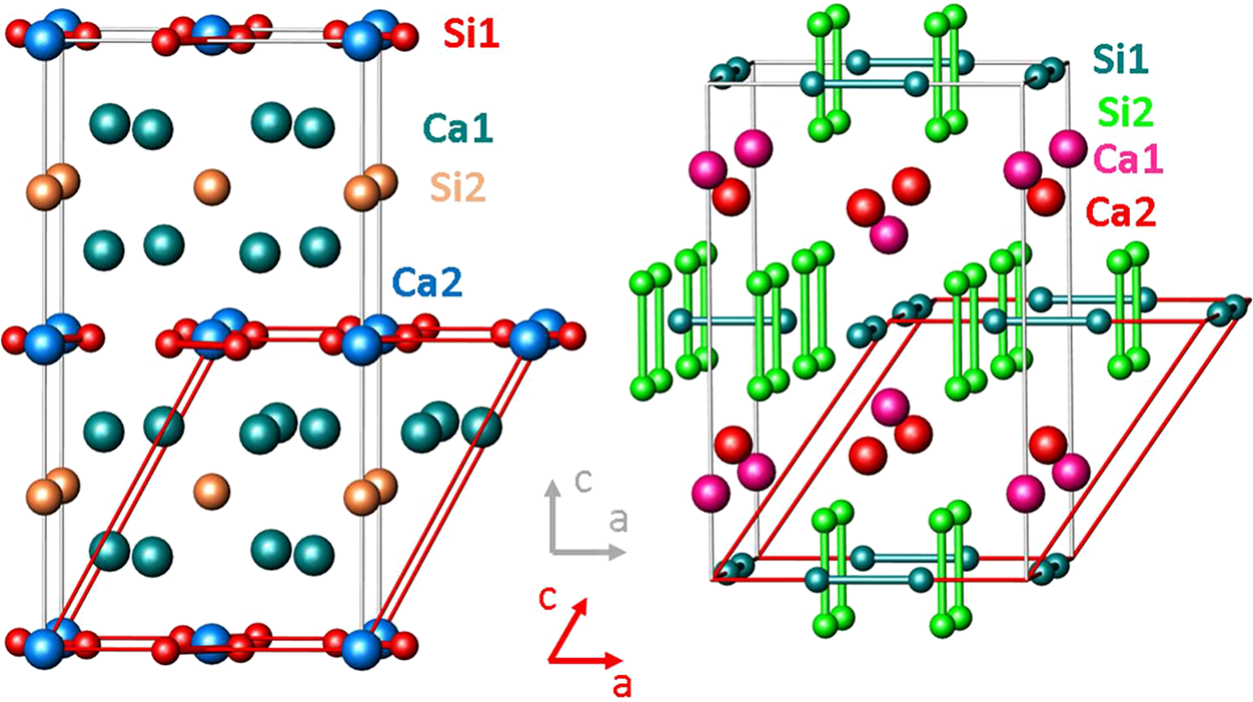
Crystal structures of (left) Ca_5_Si_3_ and (right) CaSi_3_. In the in the electron-precise Zintl phase Ca_5_Si_3_, silicon forms isolated Si anions, which are engirded by cationic calcium ions, and Si_2_ dumbbells.^36^ In metallic CaSi_3_, two different Si_2_ units adopt perpendicular orientations.^46^ The bond distances *d*(Si–Si) within the dumbbells (red) amount to 2.42 Å in Ca_5_Si_3_ and to 2.390(1) Å (turquoise) and 2.4037(8) Å (green), respectively, in CaSi_3_. Within the layers of the covalent metal, there are additionally four longer Si–Si contacts per silicon atom of 2.5839(5) Å and 2.7037(6) Å, respectively. The crystallographic unit cell is shown in gray, and red lines indicate the primitive repeat unit used for the calculations.

The depicted primitive unit cells used for the solid-state computations of Ca_5_Si_3_ and CaSi_3_ contain two and four formula units, respectively (Figure 1). The following analysis is based on these entities holding the same total number of atoms. According to the Zintl concept, the electron count for two formula units Ca_5_Si_3_ yields (Ca1^2+^)_8_(Ca2^2+^)_2_[(Si1^3–^)_2_]_2_(Si2^4–^)_2_ and, thus, 44 valence electrons. Assuming complete charge transfer from the calcium atoms, the 6 silicon anions hold a total of 12 electrons in 3s and 32 in 3p states. The primitive unit cell of CaSi_3_ holds four formula units, Ca_4_Si_12_, with a total of 56 electrons. In the covalent metal, 8 electrons stem from Ca 4s, and the remaining 48 e from silicon states. Out of those, 24 have originally Si 3s and another 24 Si 3p character.

### Density of States and Electron Density

**Figure 2:**
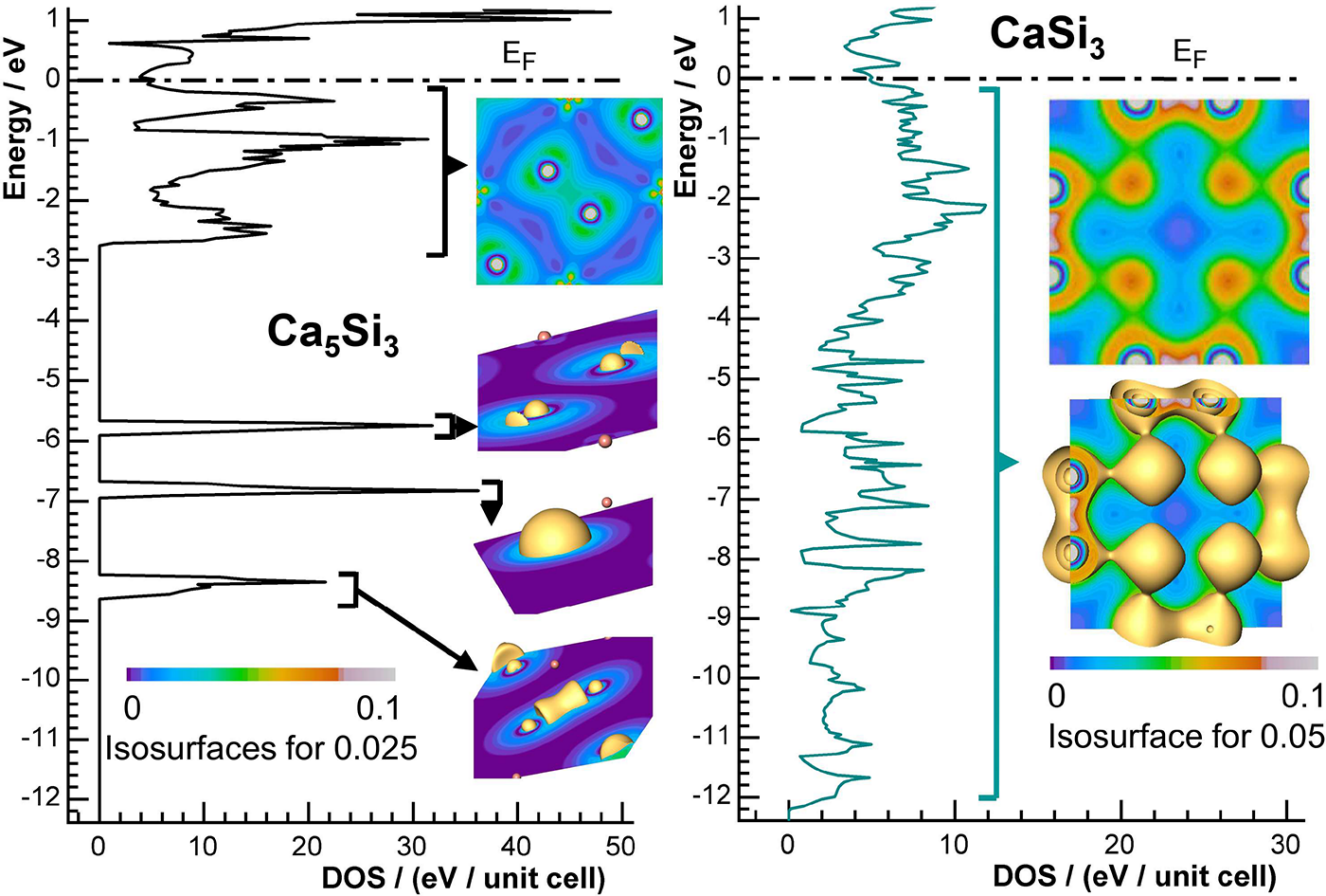
Calculated density of states (DOS) and electron density for (left) Ca_5_Si_3_ and (right) CaSi_3_. The DOS of the Zintl-phase Ca_5_Si_3_ exhibits localized bands, which are typical features for solids comprising weakly interacting building units. Nevertheless, there is a region with large dispersion, in which a clear separation is no longer possible since different states contribute within the given energy window. This region, located between −2.7 eV and E_F_, indicates metallic behavior of the compound. The DOS for CaSi_3_ shows a continuous distribution over the complete range of valence states, signaling metallic character of the phase. Insets show the electron densities, which are calculated from the states in the indicated energy ranges.

The calculated electronic density of states (DOS) for Ca_5_Si_3_ and CaSi_3_ (Figure 2) shows pseudogaps around the Fermi level for both compounds. The considerable number of states at the Fermi level clearly indicates the metallic character of both compounds. Despite this similarity, the compounds exhibit fundamental differences of their electronic structures.

Ca_5_Si_3_ exhibits clearly localized states at −8.4 eV, −6.8 eV, and −5.8 eV, a finding in agreement with a previous study.^52^ In addition, there is a significantly structured region just below the Fermi level. Localized states like those in Ca_5_Si_3_, are typically formed by a single type of orbital that hardly mixes with states of other neighboring atoms. Consequently, they show little dispersion in the corresponding energy window. In real space, this is reflected by a localized electron distribution as calculated from the states in the respective energy range (insets of Figure 2). The lowest energy window around −8.4 eV captures states that contribute to the bonding Si1–Si1 interaction. Later, we will show that this density belongs to the σ-bonding states formed by Si1 3s orbitals.

The electron density is high between the Si atoms because of constructive orbital overlap. The antibonding component is formed by the states around −5.8 eV. The corresponding electron density is zero at the bond midpoint of two silicon atoms. Shortly, we will show that this energy range is formed by the σ*-states, the antibonding combinations of Si 3s orbitals. Finally, the localized states around −6.8 eV belong to 3s states of the isolated Si2 ions. As these show no significant covalent interactions, the associated density of states does not split and the related electron density remains almost spherically symmetric. The states from −2.7 eV to E_F_ exhibit significant dispersion and form a structured, yet delocalized band.

In case of the electron-precise Ca_5_Si_3_, the dispersed band holds a total of 32 electrons, which mainly originate from Si 3p and Ca states as we will show soon. The section of the computed electron density for this energy range exhibits contributions close to the Si1 dumbbells as well as elevated values of electron density at the calcium atoms.

Per contra to the situation in the electron-precise Zintl phase, the density of states for the covalent metal CaSi_3_ (Figure 2) shows prominent dispersion involving the states of all 56 valence electrons. The dispersive band ranges from −12.2 eV to the Fermi level. The corresponding real-space representation of the electron density prominently depicts discrete (Si1)_2_ dumbbells parallel to the *a* or *b* axes of the crystallographic body centered unit cell (directions ⟨100⟩). The high values close to the center originate from the perpendicular (Si2)_2_ units oriented parallel to the *c* axis (direction [001]). Note that the computed electron density associated to the bands with broad dispersion is much higher in CaSi_3_ than in Ca_5_Si_3_. With the same number of atoms in the unit cell, the regions represent 56 electrons in case of CaSi_3_ and only 32 electrons in case of Ca_5_Si_3_.

The electron density of Ca_5_Si_3_, which is computed for the dispersive bands below the Fermi level, is strongly structured, with charge being concentrated on the silicon dumbbells and deep valleys between those fragments. In the covalent metal CaSi_3_, the distribution is clearly smoother with significant density between the dumbbells. In agreement with an earlier study on the nearby element aluminum,^54^ we assign this finding to a more pronounced metallic character of the anionic building units of CaSi_3_ as also revealed by the uninterrupted DOS of the valence electrons.

### Partial Density of States and Partial Electron Densities

The concept of dividing the total density of states into atom- and orbital-resolved contributions is a well-established method for shedding light onto the interactions between individual groups or atoms in solids.^55,56^

Aim of the method is the projection of crystal orbitals, which are confined within the unit cell, onto an atomic orbital basis for an intuitive chemical interpretation. The corresponding procedure is applicable to real space computations. While the total electron density is computed from contributions of all crystal orbitals, expansion of those crystal orbitals in an atomic basis yields individual orbital contributions. For characterizing the interaction between two atoms, the unified and the intersection density are of special interest. The unification is a measure for the contribution of the chosen orbital set (usually two orbitals on different atomic sites). The intersection samples only the cross terms for a chosen orbital set and provides information on the nature of the interaction in position space. Positive values indicate a bonding, negative ones an antibonding interaction. Consequently, values around zero represent noninteracting orbitals. For details, see the Theoretical Basis section.

**Figure 3:**
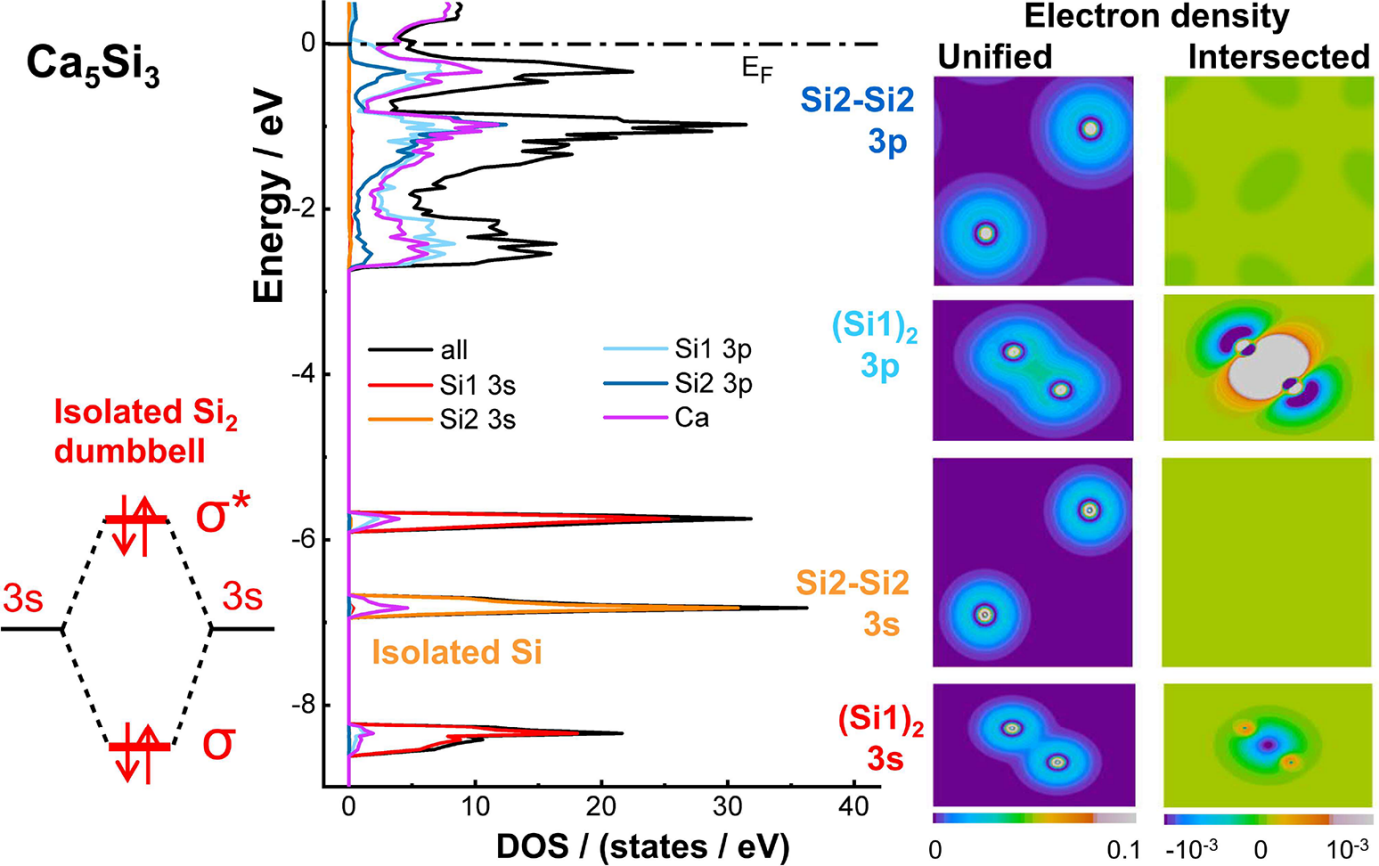
Chemical bonding in Ca_5_Si_3_. (outer left) Schematic molecular orbital diagram in the region of the 3s states for a diatomic silicon dumbbell. (middle left) Calculated density of states for Ca_5_Si_3_ and selected partial contributions. (middle right) Unified and (outer right) intersected electron density. Depicted are partial electron densities of the indicated atoms and orbital contributions.

In accordance with an earlier independent study,^52^ the partial density of states for Ca_5_Si_3_ (Figure 3) indicates that the localized states at −8.4 eV and −5.8 eV originate mainly from the 3s orbitals of the Si1 atoms within the dumbbells. The separation into an upper and lower band is assigned to the interaction of the two silicon atoms within the dumbbell, which is similar to the situation in molecular diatomic fragments. Since both bands are strongly confined in energy, the molecular orbital scheme for isolated entities may serve as a suitable model for approaching the chemical bonding in the solid. The 3s orbitals of the Si1 atoms form two bands, one with bonding and one with antibonding character, similar to the σ and σ* states of a molecular orbital scheme. As both bands are fully occupied, the resulting net interaction is slightly antibonding. This aspect also shows in the partial density in real space. The unified electron density computed from the 3s orbitals of two neighboring silicon atoms in a dumbbell basically represents the shape of the constituting 3s orbitals. The intersection density is negative (blue region) around the midpoint of the tie line between the Si1 atoms. The regions of positive values (shown in yellow and orange) close to the nuclei are a consequence of the nodes of the 3s orbitals. In total, however, the net interaction is negative, therefore indicating antibonding character (Table 1) as the regions of negative values overcompensate the positive ones. With increasing distance to the dumbbell, the intersection density tends toward zero as both orbitals decay quickly.

**Table 1:** Electron Density Per Atomic Orbital Contribution of Silicon Atoms or Dumbbells in Ca_5_Si_3_ and CaSi_3_; The Total Density Corresponds to the Sum of the Orbital Density, and the Intersected Density Indicates Bonding (Positive Values) or Antibonding (Negative Values) Character of the Interaction

Atoms and states	Sum orbital density	Sum of the norms	Intersected density
**Ca**_**5**_**Si**_**3**_			
dumbbell (Si1)_2_			
Si1–Si1 3s	2.381	2 × 1.206	–0.032
Si1–Si1 3p	5.176	2 × 2.496	+0.184
isolated Si2			
Si2–Si2 3s	2.575	2 × 1.287	0.000
Si2–Si2 3p	5.127	2 × 2.571	–0.015
**CaSi**_**3**_			
dumbbell (Si1)_2_			
Si1–Si1 3s	2.253	2 × 1.136	–0.018
Si1–Si1 3p	3.876	2 × 1.865	+0.014
Si1–Si1 3d	0.122	2 × 0.059	+0.004
dumbbell (Si2)_2_			
Si2–Si2 3s	2.409	2 × 1.214	–0.019
Si2–Si2 3p	3.643	2 × 1.749	+0.146
Si2–Si2 3d	0.111	2 × 0.052	+ 0.007

By contrast, there is no indication for significant interactions of the 3s orbitals of neighboring isolated Si2 ions. This is indicated in the partial density of states density by a single peak holding the 3s states, a finding in accordance with a previous study. Consequently, the intersection density is essentially zero (light green). Although the 3p orbitals of Si2 do overlap slightly because they are more extended in space, the resulting interaction is slightly negative (dark green).

A different situation is found for the 3p orbitals of the Si1 dumbbell atoms in Ca_5_Si_3_. They interact intensely as signalized by the large dispersion of the partial DOS (−2.7 eV to E_F_, light blue curve) and the respective real space representations. The unified density for the 3p orbitals of two neighboring Si1 dumbbell atoms differs largely from that of a simple superposition of noninteracting atoms. The electron distribution is pronouncedly higher around the center of the tie line between the atoms than in other regions with the same distance to the nuclei. The intersection density visualizes the aspect even more clearly. The high positive values of the intersection density between the dumbbell atoms (white region) exceed the selected scale range, thus evidencing strong covalent chemical bonding by means of the 3p orbitals of the Si1 atoms in the dumbbells.

**Figure 4:**
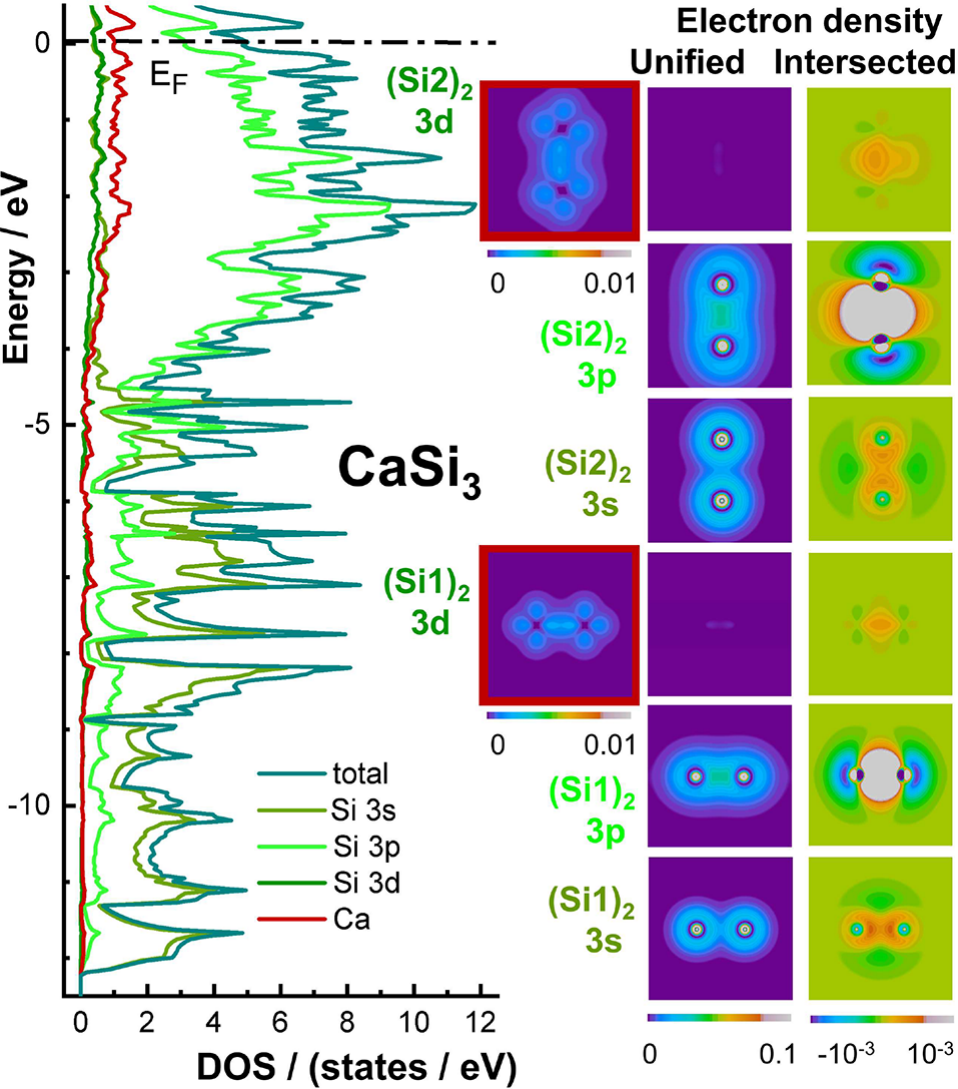
Chemical bonding in CaSi_3_. (left) Calculated density of states. (middle) Unified and (right) intersected electron density distribution. Shown are partial electron densities referring to the indicated atoms and orbital contributions.

In Ca_5_Si_3_, in which the Si2 ions and the (Si1)_2_ units are separated by calcium atoms, the 3s orbitals form rather localized bands whereas the dumbbells in CaSi_3_ constitute condensed layers involving secondary Si–Si contacts. Accordingly, the dispersion of the DOS is extremely pronounced and involves all valence electrons including the 3s states of silicon (Figure 4). We will show in the next section that it is exactly this spread of the Si 3s states which additionally strengthens the Si–Si bonds in CaSi_3_. In the covalent metal, the real space representation of the density computed from the 3p contributions of Si1–Si1 and Si2–Si2 are similar to those of the dumbbell atoms in the Zintl phase. High values of the unified electron density (orange to white) point at strong bonding between the atoms within the Si_2_ species. The intersection density shows clearly highly positive regions around the midpoints between neighboring atoms (white region). Thus, the 3p orbitals are strongly bonding in CaSi_3_, but they show a much larger dispersion than in Ca_5_Si_3_. In addition, we find a small, but significant contribution of Si 3d orbitals to the bonding in CaSi_3_, which is clearly absent in the electron-precise Zintl phase. The total amount of the 3d contribution is small, but the positive values of the intersecting density clearly emphasize their bonding nature.

Another striking difference is observed for the 3s orbitals of silicon. Their intersection densities in CaSi_3_ show positive values (indicated in orange) and, thus, bonding between the dumbbell atoms. By contrast, Ca_5_Si_3_ features a net antibonding interaction of the 3s states. Nevertheless, the presence of two separate maxima for the intersection density in CaSi_3_ denote that there are also some antibonding 3s contributions. The minimum in the middle of the bonding region is determined by these states, but their sum is too small to fully compensate the 3s bonding contributions. Therefore, the net interaction of the Si 3s orbitals is bonding in CaSi_3_.

### Quantitative Electron Count

**Figure 5:**
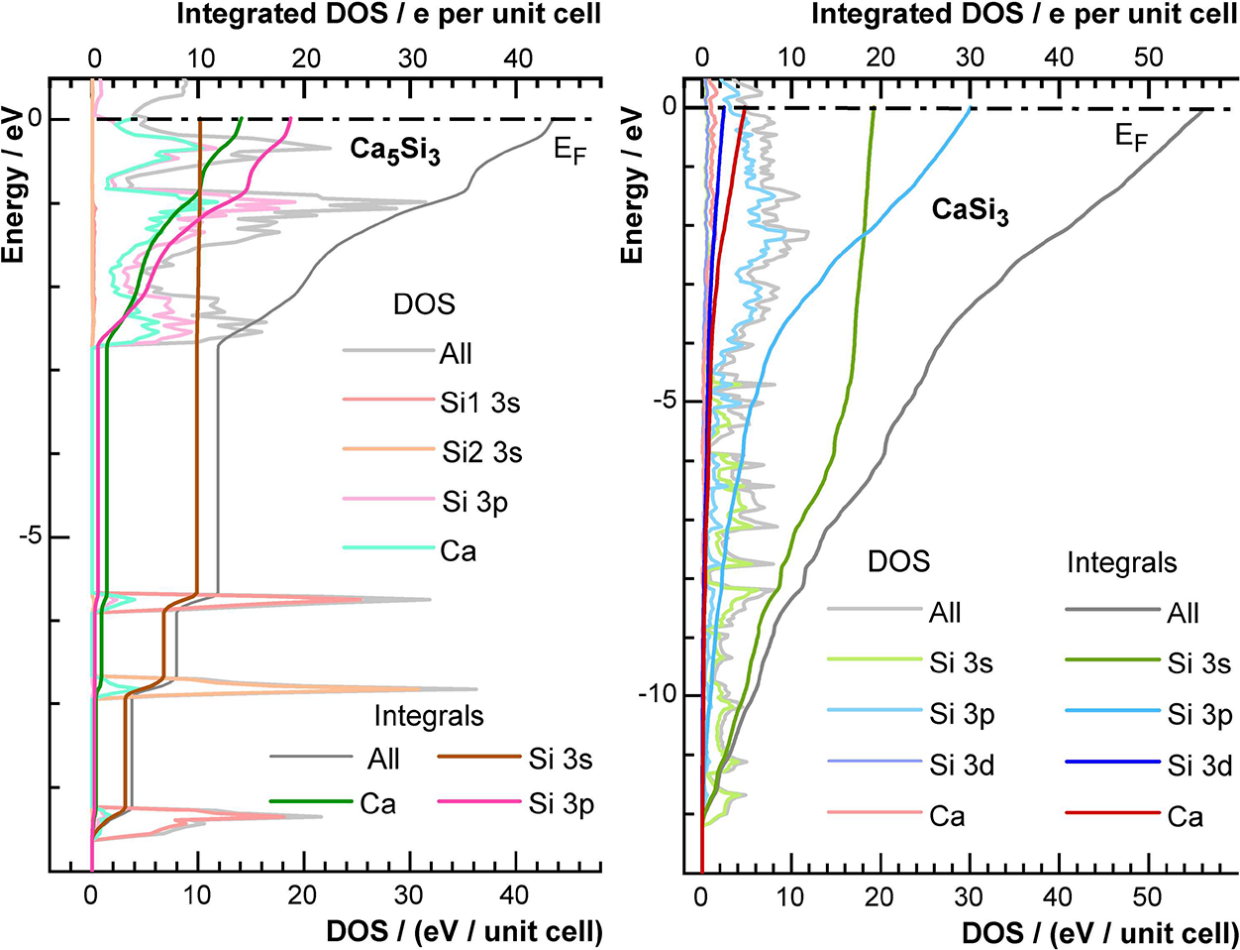
Calculated density of states and integrated DOS of (left) Ca_5_Si_3_ and (right) CaSi_3_. The integrated values of the Zintl-phase show four electrons for each localized band, resulting in a stepwise behavior of the integral. The region with large dispersion between −2.7 eV and E_F_ contains the remaining 32 electrons and shows a steady increase of the number of states. The DOS for CaSi_3_ shows a continuous distribution, and the integrated value for the number at the Fermi level amounts to 56 for the primitive unit cell.

In order to quantify the presented qualitative bonding analysis, DOS, p-DOS, and their successive sums up to E_F_ are evaluated. In Ca_5_Si_3_ with two formula units in the primitive unit cell, calcium and silicon occupy two different positions each, and the composition may be expressed as (Ca1)_8_(Ca2)_2_[(Si1)_2_]_2_(Si2)_2_, resulting in 44 electrons in total. As the total density of states integrates to the same number (Figure 5, gray curve), the energy range extending from −8.6 eV to E_F_ captures the valence electrons completely. The three localized bands between −8.6 eV and −5.6 eV integrate to four electrons each (gray curve), 12 in total, and the delocalized broad band above −2.7 eV holds the remaining 32 electrons. As shown in the last section, the localized bands at −8.4 eV and −5.8 eV mainly result from Si1 3s orbitals located at the silicon dumbbells (red curve in Figure 5) and can, therefore, be described as nearly fully occupied bonding and antibonding states of a silicon dimer. As a consequence, the net 3s interaction is repulsive, which is concordantly reproduced by the negative value for the integral of the intersection density (see Table 1). The two isolated anions of Si2 hold four electrons in a single localized 3s band, and the value of zero for the integrated intersection density is in line with the absence of significant interactions.

For the 3p states of Si2, a negative value of the intersection integral signals antibonding character as well. In total, the anions formed by Si2 are best described as isolated entities with purely ionic interactions. In contrast, strong covalent bonding is signified by the positive value for the 3p orbitals of the dumbbell atoms Si1. In agreement with a simple orbital picture, the integrated numbers imply that the 3p orbitals act as acceptors in the charge transfer process from calcium to silicon. The population number of the calcium amounts to 14 (11 for Ca1 and 3 for Ca2) in comparison to the sum of 20 valence electrons for the neutral atoms. We like to note here that the charge transfer, which is estimated from band structure calculations, is usually much smaller than the numbers resulting from the oxidation states, which are based upon the assumption of complete charge transfer. In total, two Si2 ions hold six 3p electrons, which amounts to a charge transfer of one electron each. Four Si1 atoms in dumbbells hold 12 3p electrons, again resulting in a charge transfer of one electron from the Ca atoms to each Si1 anion. The finding that the donated electrons as a whole occupy 3p states of Si is a fundamental difference to CaSi_3_ and substantially affects the bonding properties as will be shown next.

A second important difference between Ca_5_Si_3_ and CaSi_3_ is the significant population of silicon and calcium 3d orbitals in the covalent metal. This finding is in accordance with an earlier study on calcium silicides and the more recent diligent analysis of CaSi specifying significant 3d contributions of calcium.^50,38^ However, another marked and even more essential difference of the covalent metal in comparison to the Zintl phase concerns the energetic rearrangement of the 3s and 3p orbitals of silicon.

In CaSi_3_, there are four formula units in the primitive unit cell. Again, calcium and silicon occupy two different positions each, and the composition may be sketched as (Ca1)_2_(Ca2)_2_[(Si1)_2_]_2_[(Si2)_2_]_4_, resulting in 56 electrons in sum. The pronounced dispersion of the states (see Figure 5), including those formed by silicon 3s and 3p, goes along with a spread of the σ*-like states above the Fermi level. As a consequence, the 12 silicon atoms of the primitive unit cell hold a total of only 18 electrons with 3s character (green curve). With six electrons promoted to mainly silicon 3p states, the total yield of these amounts to 30 (light blue curve). In a local picture for the silicon dumbbells in CaSi_3_, full occupation of the bonding and antibonding states originating from 12 Si 3s orbitals would result in null bonding contribution. Consequently, the promotion of six electrons strengthens the bonding interaction by an effective depletion of antibonding σ* states. With regards to the 3p states, the bonding π-type orbitals have capacity for 36 electrons while being occupied with 30. The stabilizing effect of the redistribution becomes also apparent in the values for the intersection integrals of 3s, 3p, and 3d contributions for the Si1–Si1 and Si2–Si2 interactions. Thus, the promotion of six electrons with Si 3s character into Si 3p orbitals has the effect of stabilizing the covalent bonds in the Si_2_ units of CaSi_3_ while simultaneously contributing to metallic bonding.

### Electron Localizability Indicator ELI-D

In the previous sections, we analyzed partial contributions for the Si dumbbells in Ca_5_Si_3_ and CaSi_3_. The analyses based on atomic orbital contributions conducted so far point to stronger bonding within the Si dumbbells in the covalent metal CaSi_3_ compared to those in the Zintl phase Ca_5_Si_3_. This aspect should also show in chemical bonding analysis in real space.

**Figure 6:**
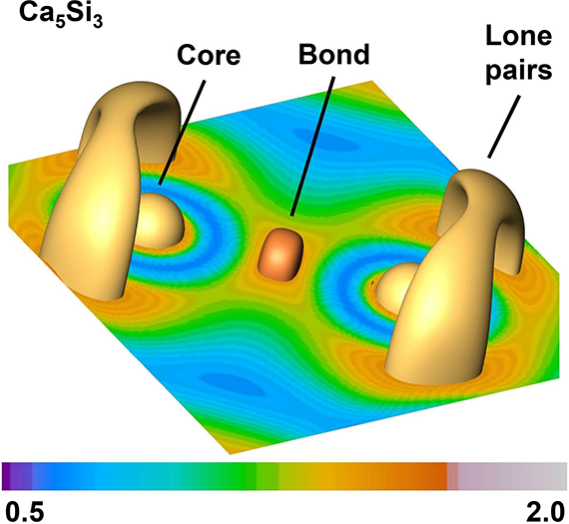
ELI-D distribution of the dumbbells in Ca_5_Si_3_. Shown are an orthoslice and isosurfaces for the value of 1.53 au (yellow) indicating core and lone pair region. The irreducible domain for the bonding region is shown with a value of 1.4 au in orange.

**Figure 7:**
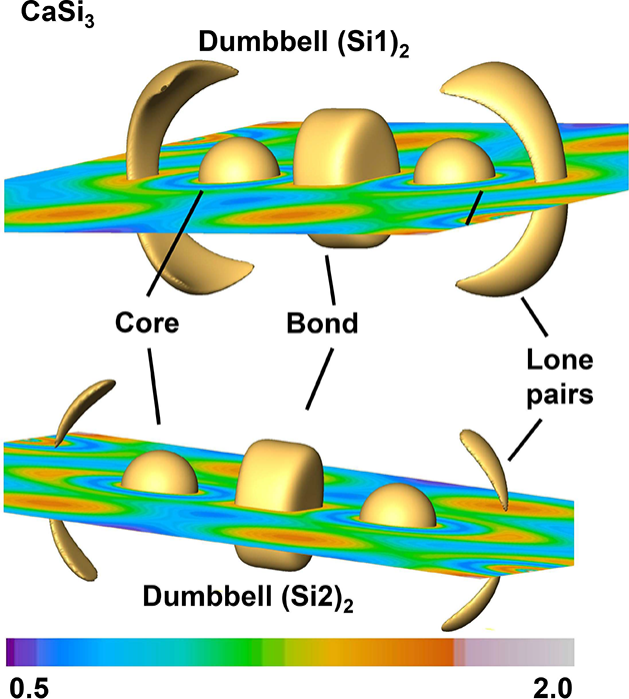
ELI-D distribution of the two types of dumbbells in CaSi_3_. Shown are orthoslices and isosurfaces (yellow) for the values (top) 1.41 au and (bottom) 1.435 au indicating core, lone pair, and bonding regions.

The electron localizability indicator (ELI-D) is a suitable measure for this purpose. In contrast to atomic orbital analysis, ELI-D is based on the wave function involving all orbital contributions. As such, ELI-D is invariant under unitary rotation of the wave function (which is an important aspect in quantum mechanics and applies to all measurable quantities). ELI-D compares the number of same-spin pairs with the number of electrons in small, mutually exclusive, space-filling regions called microcells. Thus, ELI-D provides a measure for the Fermi correlation in position space. It has been shown that this local measure relates to the chemically intuitive bonding picture by dividing the total space into chemically meaningful sections like core, lone pair, and bonding regions.^57,58^ The ELI-D pictures of the dumbbells in Ca_5_Si_3_ (Figure 6) and CaSi_3_ (Figure 7) clearly illustrate suchlike typical separations for the covalently bonded diatomic Si_2_ species. Beyond that, electron counts over such regions by integration in so-called basins often provide numbers in qualitative agreement with classical counting rules. Therefore, we use ELI-D here to also test our previous assumption about the bond strengthening in the Si dumbbells of CaSi_3_.

**Figure 8:**
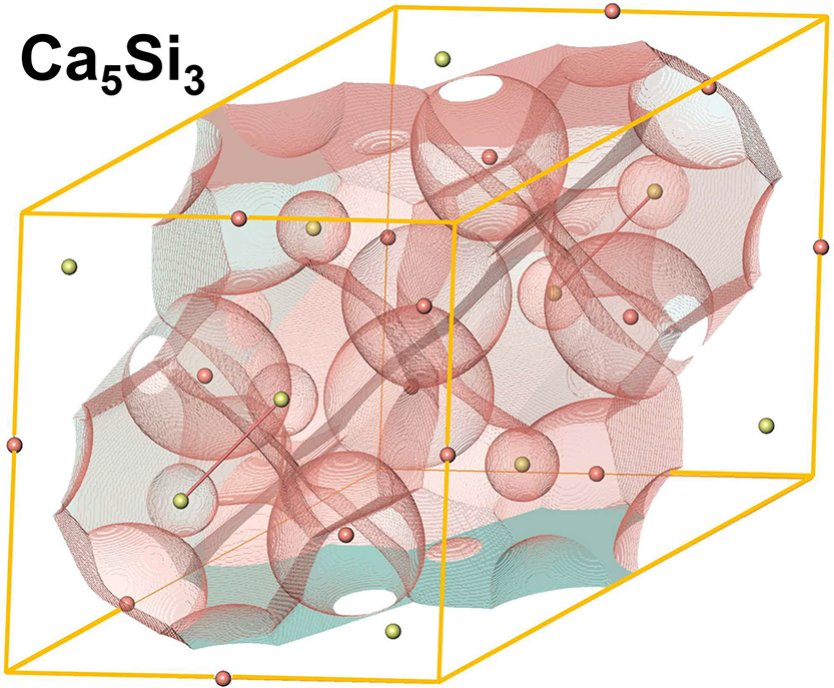
Calculated electron localizability indicator (ELI-D) specifying the basins of Ca_5_Si_3_. The visualization illustrates the exhaustive space filling nature of the separation with close resemblance to the classical chemical model separating core and valence regions. Orange lines indicate the primitive unit cell used for the computations.

The ELI-D basins (boundaries shown in transparent rose) for Ca_5_Si_3_ (Figure 8) provide a binary separation of the total space which is exhaustive, i.e., space-filling and nonoverlapping. This property is contrary to orbital separation schemes, which are fuzzy so that one point in space may belong partially to more than one atom. Thus, ELI-D yields a natural chemically meaningful division of space and enables counts of the electron populations. Electron balances (Tables 2 and 3) are obtained by integrating the electron density in the respective ELI-D basins.

**Table 2:** Electron Population for the ELI-D Basins of Ca_5_Si_3_

Ca_5_Si_3_	Calcium	Isolated Si	Si–Si dumbbell
Inner core	10.1	10.1	2 × 10.1
Outer shell	8.4	7.0	2 × 5.5 lone pair
			1.3 bond
Total	18.5	17.1	32.3
Net charge	+1.5	–3.1	–4.3

**Table 3:** Electron Population for the ELI-D Basins of CaSi_3_

	Calcium	Si1–Si1 dumbbell	Si2–Si2 dumbbell
Inner core	10.1	2 × 10.1	2 × 10.1
Outer shell	8.4	2 × 3.5 lone pairs	2 × 3.6 lone pairs
		1.9 bond	1.6 bond
Total	18.5	29.1	29
Net charge	+1.5	–1.1	–1.0

The inner core regions comprise the first and the second atomic shell, and thus, contain 10.1 electrons in all cases. The respective value for the outermost shell of calcium amounts to 8.4 electrons. Therefore, the charge transfer from each calcium to the silicon atoms amounts to 1.5 electrons for both compounds. Note that computed charges from quantum mechanical calculations are usually smaller than our chemical model since a complete charge transfer is energetically not favorable, but electrons spread into the regions of other atoms. Yet, the value is still a rather high charge transfer and can, to a good extent, be related with our chemical picture of a double-charged calcium cation. Similar findings apply to the isolated silicon ion in Ca_5_Si_3_. With 7.0 electrons in the third shell, the final charge sums up to −3.1. In a simple chemical picture, this fragment relates to Si^4–^. The lone pair basins of the Si dumbbells contain 5.5 and the respective bonding basin 1.3 electrons. Although the complete charge transfer of −4.3 electrons is considerably smaller than −6, the population of the lone pairs and the electron count for the bonding region point to a comparison with the molecular fragment Si_2_^6–^ with a single bond and three lone pairs for each Si atom rather than to the situation found in the MO scheme. Thus, we suggest that Ca_5_Si_3_ is essentially being built from the classical chemical fragments (Ca^2+^)_5_(Si2)^4–^(Si1)_2_^6–^, implying strong ionic interactions between silicon and calcium. However, in line with the considerable amount of metallic bonding in the solid, the charge transfer between the fragments is reduced. As a consequence, the electronic population in the respective outermost Si basins becomes smaller, finally yielding the fragments Ca^1.5+^, Si2^3.1–^, and Si1_2_^4.3–^ in close resemblance to classical fragments.

The situation for CaSi_3_ (Figure 8) is by far more complex. Again, we find the same core populations and also the charge transfer from calcium to silicon is the same with 1.5 electrons. The two different types of Si_2_ dumbbells capture 3.5 electrons and 3.6 electrons in their respective lone pair regions as well as 1.9 and 1.6 electrons in the bonding basin, respectively. While the number of lone pair electrons with 3.5 seems to imply the molecular fragment Si_2_^4–^, the total number of electrons in the system is not sufficient to fill the respective MO scheme. Even in case of a hypothetical complete charge transfer from Ca to Si, the system has to adopt to this electron deficiency and consequently forms a metallic state. In accordance with the metallic character of the system, the covalent metal CaSi_3_ is therefore closer packed than its electron precise counterpart Ca_5_Si_3_. The densities come to 2.76 and 2.18 g/cm^3^ for CaSi_3_ and Ca_5_Si_3_, respectively.

As in the case of atoms, the driving force to form a metallic assembly is, thus, the electron deficiency of the molecular building units in CaSi_3_. The Si–Si bond of the Si dumbbells profits from the collective metallic bonding due to the spread of the s-states and thus, the depopulation of antibonding σ* and π* orbitals compared to the bonding situation in the molecular fragments of Ca_5_Si_3_. Consequently, the ELI-D bonding basins in CaSi_3_ reflect the stronger bonding by capturing more electrons than the corresponding Si–Si bonding basin in Ca_5_Si_3_.

## Conclusion

The present study demonstrates that the silicon dumbbells in the Zintl phase Ca_5_Si_3_ and the covalent metal CaSi_3_ may be described as ionic, molecular fragments, in which the chemical bonding is largely in accordance with classical chemical concepts despite a considerable amount of metallic interactions. Ca_5_Si_3_ features a combination of localized 3s states of silicon and metallic dispersion just below the Fermi level despite its electron-precise character. Topological analysis of the electron localizability indicator (ELI-D) confirms that the calculated charges of the calcium atoms as well as those of the isolated silicon anion and the dumbbells are significantly smaller than those predicted on the basis of the simple oxidation states. In the covalent metal CaSi_3_, the large energy spread of the silicon 3s states combined with the redistribution of electrons from Si 3s into 3p states has the effect of strengthening the bonding interactions within the diatomic anions. Consequently, CaSi_3_ shows a higher electronic population in the ELI-D bonding basins of the dumbbells than Ca_5_Si_3_.

## COMPUTATIONAL SECTION

Quantum mechanical calculations for Ca_5_Si_3_ and CaSi_3_ were performed with the FPLO package and the FHI-aims program using the experimentally obtained crystallographic unit cells and position parameters for a closed-shell calculation on a scalar relativistic level using tight basis sets and a mesh of 6 × 6 × 6 and 10 × 10 × 10 of *k*-points in the Brillouin zone for the FHI-aims and FPLO calculations, respectively.^59,60^ Both codes were modified in order to employ the bifunctional FB16.^61^ Subsequent real space analyses were performed with a mesh of 0.05 bohr using the program DGrid.^62^

## THEORETICAL BASIS

For characterizing the interaction between two atoms, two types of partial densities have been employed. The unified electron density ρ^∪^ for two orbitals *m* and *n* located at two atoms *A* and *B* computed for orthonormal orbitals is given by
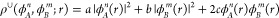
where ϕ_*A*_^*n*^(*r*) is the *n*-th orbital located at atom *A*, ϕ_*B*_^*m*^(*r*) is the *m*-th orbital located at atom *B*, and *a*, *b*, and *c* are parameters that have been determined by the quantum mechanical calculation. It is important to note that *a* and *b* are always non-negative numbers because they result from a square of a real number. The possible values of *c* equal zero for noninteracting atoms, and positive or negative values indicate bonding or antibonding interactions, respectively. Since ϕ_*A*_^*n*^(*r*) and ϕ_*B*_^*m*^(*r*) are functions in position space, which may adopt positive or negative values, the same holds for the unified density ρ^∪^(*r*). In practice, however, the value of ρ^∪^(*r*) is dominated by the first two terms in the equation as the sum of the norms of the products is usually bigger than the value of ϕ_*A*_^*n*^(*r*) ϕ_*B*_^*m*^(*r*), especially when the considered orbitals are located on different atoms. Therefore, the density computed as the intersection between ϕ_*A*_^*n*^(*r*) and ϕ_*B*_^*m*^(*r*) is often the more suitable quantity for revealing the type of interaction between the corresponding atoms.

The intersected density is computed as
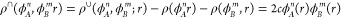
The integral over the intersection density yields a net number of charges *q*^∩^
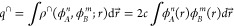
which accounts for the resulting net interaction. Values *q*^∩^ > 0 indicate a bonding, *q*^∩^ < 0 an antibonding, and *q*^∩^ = 0 the absence of interaction. In addition, the local distribution of *ρ*^*n*^(*r*) provides information on the local distribution of bonding, non- or antibonding interactions.
